# Dynamics of circulating endothelial cells and endothelial progenitor cells in breast cancer patients receiving cytotoxic chemotherapy

**DOI:** 10.1186/1471-2407-12-620

**Published:** 2012-12-26

**Authors:** Yu-Hsuan Kuo, Ching-Hung Lin, Wen-Yi Shau, Te-Jung Chen, Shih-Hung Yang, Shu-Min Huang, Chun Hsu, Yen-Shen Lu, Ann-Lii Cheng

**Affiliations:** 1 Department of Oncology, Chi-Mei Hospital, Tainan, Taiwan; 2 Department of Oncology, National Taiwan University Hospital, No. 7, Chung-Shan South Road, Taipei, 10002, Taiwan; 3 Center For Drug Evaluation, Division of Health Technology Assessment, Taipei, Taiwan; 4 Oncology Translational Research Center, TTY Biopharm Company Limited, Taipei, Taiwan; 5 Departments of Internal Medicine and Oncology, National Taiwan University Hospital, 7 Chung-Shan South Road, Taipei, 10002, Taiwan

**Keywords:** Circulating endothelial cells, Endothelial progenitor cells, Breast cancer, Angiogenesis, Biomarkers

## Abstract

**Background:**

The abundance of circulating endothelial cells (CECs) and circulating endothelial progenitor cells (CEPs), which serve as surrogate markers for angiogenesis, may be affected by chemotherapy. We studied their dynamic change during consecutive cycles of chemotherapy.

**Methods:**

We collected blood samples from 15 breast cancer patients, who received a total of 56 courses of systemic chemotherapy, and measured the CECs, viable CECs (V-CECs), and CEPs by six-color flow cytometry within the seven days prior to chemotherapy, twice a week during the first and second cycles of chemotherapy, and then once a week during the subsequent cycles.

**Results:**

The CEC, V-CEC, and CEP levels all significantly decreased from day 1 of treatment to the first week of chemotherapy. After one week of chemotherapy, the CEC and V-CEC levels returned to a level similar to day 1. The CEP level remained significantly reduced after the first week of chemotherapy, but gradually rebounded until the next course of chemotherapy. After six cycles of chemotherapy, the total number of CEC and V-CEC cells trended toward a decrease and the CEP cells toward an increase. Clinical factors, including the existence of a tumor, chemotherapy regimens, and the use of granulocyte colony stimulating factor, did not significantly affect these results.

**Conclusions:**

The CEC and CEP counts change dynamically during each course of chemotherapy and after the chemotherapy cycles, providing background data for any future study planning to use CECs and CEPs as surrogate markers of angiogenesis in antiangiogenesis treatments combined with chemotherapy.

## Background

Antiangiogenic drugs play an important role in the current treatment of cancer. However, single-agent antiangiogenesis therapy has had only a modest effect except in renal cell carcinoma. The combination of antiangiogenesis drugs and cytotoxic chemotherapy has been approved for colon, lung, and breast cancers [1-7]. Chemotherapy with the addition of bevacizumab, an antivascular endothelial growth factor (VEGF) antibody, significantly improves the overall survival of patients with metastatic colorectal cancer [1,3,4,6-8]. Bevacizumab can also improve the objective response rate and progression-free survival of breast and lung cancer patients treated with chemotherapy [9-18].

Although antiangiogenesis treatments are widely used, we still lack a surrogate marker to select the populations for whom the drug will be effective and to define the optimal biological dose and treatment timing for antiangiogenic therapy. Currently, the antiangiogenesis markers that have been extensively studied include functional imaging, serum angiogenesis-related markers, and measuring circulating endothelial cells (CECs).

The level of CECs, which are derived from the turnover of cells in the blood vessel wall, increases in certain cancers [19-25]. Circulating endothelial progenitor cells (CEPs), a subpopulation of CECs, have a progenitor-like phenotype, are derived from bone marrow, and contribute to the vasculogenesis of late-stage cancer [26-30]. Shaked et al. [31] demonstrated that CECs and CEPs could serve as surrogate markers to define the optimal biological dose in antiangiogenesis therapy. The baseline levels of CEC and CEP and the changes in their levels from pretreatment to post-treatment may serve as pharmacodynamic biomarkers to predict responses to antiangiogenesis therapy and to metronomic chemotherapy [31-35]. However, animal studies have shown that CEP mobilization is induced by treatment with the maximal tolerated dose (MTD) of chemotherapy, such as taxane and fluorouracil within a few days after the chemotherapy [36,37]. Preventing CEP mobilization with anti-VEGFR2 blocking antibodies could result in enhanced antitumor effects [37]. Therefore, the timing of the measurement of the CECs and CEPS is important when using them as surrogate markers, especially when antiangiogenesis therapy is combined with MTD chemotherapy. To clarify the dynamic pattern of CECs and CEPs, we designed a study to describe the detailed dynamic change of CECs and CEPs during each cycle of chemotherapy treatment.

## Methods

### Patients

Our study was approved by the Research Ethics Committee of National Taiwan University Hospital. We enrolled 15 breast cancer patients who received systemic chemotherapy in the form of neoadjuvant, adjuvant, or palliative chemotherapy. The patients were required to have histologically-confirmed breast carcinoma, to be 18 to 70 years old, to have an Eastern Cooperative Oncology Group (ECOG) performance status of less than 3, and to have hemoglobin levels above 9.0 mg/dL. Exclusion criteria were pregnancy, lactation, and uncontrolled underlying diseases, including active infections, systemic congestive heart failure, unstable angina, arrhythmia, or psychiatric disorders. All patients signed informed consents before beginning the study. Blood samples of 10 mL were drawn within the 7-day period prior to chemotherapy, then twice a week during first and second cycles of chemotherapy, and then once a week during the subsequent cycles of chemotherapy. The blood samples were processed for CEC and CEP analysis within 24 hours of collection (see below). The chemotherapy regimens and dosages depended on the doctors’ discretion.

### Biomarker evaluations

Six-color flow cytometry measured the CECs and CEPs using a method from Mancuso et al. [19,38]. Red blood cells were lysed, and then the cell suspensions were evaluated by a BD FACSCanto II cell analyzer (BD Biosciences, San Jose, CA, USA) and FACSDiva software, version 5.0.2 (BD Biosciences), with analysis gates excluding dead cells, platelets, and debris. We acquired 100,000 events per sample to analyze the percentage of CECs and CEPs. The absolute number of CECs and CEPs was then calculated by multiplying the total white cell count by the percentage of events collected in the CEC and CEP enumeration gates. We defined CECs as negative for the hematopoietic marker CD45 (BD Pharmigen, San Diego, CA) and the progenitor marker CD133 (Miltenyi Biotec, Bergisch Glabdach, Germany), and positive for the endothelial markers CD31 and CD146 (BD Pharmigen). We defined CEPs as being negative for CD45 and positive for CD31, CD146, and CD133. We differentiated viable and apoptotic cells by 7-amino actinomycin D staining [39-41]. Representative flow cytometry dot plots demonstrating the negative controls, CEC and CEP gating strategies were provided in our supplementary data. (Additional file 1: Figure S1, Additional file 2: Figure S2, Additional file 3: Figure S3, Additional file 4: Figure S4).

### Statistical analysis

Each individual patient received multiple courses of chemotherapy and was followed several times during each course of therapy. The data was two-level (patients and course of chemotherapy) repeated measurement. The changes in the levels of CEC, CEP, and viable-CEC (V-CEC) were analyzed after each course of chemotherapy by a multilevel linear mixed-effects model with random coefficients. Differences in the mean levels were considered patient-level random effects. Each patient’s chemotherapy courses were nested under the patient level as chemotherapy level. Within each chemotherapy course, the mean of the measures during the first week (day 2 to day 7), and the mean of the measures after the first week (day 8 and beyond) were compared to the measurement on the day chemotherapy started (day 1, before chemotherapy started). We analyzed the mean levels of CEC, CEP, and V-CEC for each chemotherapy course. We also analyzed how the existence of a tumor during the course of chemotherapy, the chemotherapy drug, and the use of granulocyte colony-stimulating factor (GCSF) affected these measurements. We modeled the correlation of repeated measurements within the chemotherapy courses of individual patients by first-order autoregressive error terms. Two-sided *P* values less than 0.05 were considered statistically significant. The “xtmixed” procedure from the Stata statistical package version 11 (StataCorp LP, College Station, TX, USA) analyzed the data.

## Results

### Patient characteristics

Of the 15 patients, 5 were tumor-bearing and 10 were non-tumor-bearing, with 3 (20%) receiving neoadjuvant chemotherapy, 10 (66.7%) receiving adjuvant chemotherapy, and 2 (13.3%) receiving palliative chemotherapy (Table 1). Among these fifteen patients, seven (46.7%) had GCSF administered during their courses of chemotherapy. A total of seven (46.7%) patients received an anthracycline-based chemotherapy regimen, seven (46.7%) received a taxane-based chemotherapy regimen, and two (13.3%) received other chemotherapy regimens that included vinorelbine and liposomal doxorubicin. The 15 patients received a median of four chemotherapy cycles (range: 1–6 cycles), with a total of 56 courses of chemotherapy overall in the study. The average duration between two courses of chemotherapy was 22.2 days.

**Table 1:** Patient characteristics

**Characteristic**		**No. of patients**	**%**
No. of enrolled		15	100.0%
Age, y			
	Median	56	
	Range	44-66	
Chemotherapy			
	neoadjuvant	3	20.0%
	adjuvant	10	66.7%
	palliative	2	13.3%
tumor hormone receptor			
	ER(+), PR(+)	5	33.3%
	ER(+), PR(-)	3	20.0%
	ER(-), PR(+)	1	6.7%
	ER(-), PR(-)	5	33.3%
	not applicable	1	6.7%
Her2/neu			
	Absent	5	33.3%
	1+	3	20.0%
	2+	4	26.7%
	3+	2	13.3%
	not applicable	1	6.7%
GCSF administration			
	Yes	7	46.7%
	No	8	53.3%
pT stage*			
	pT1	3	20.0%
	pT2	9	60.0%
	pT3	0	0.0%
	pT4	3	20.0%
pN stage*			
	pN0	6	40.0%
	pN1	4	26.7%
	pN2	3	20.0%
	pN3	1	6.7%
	not applicable	1	6.7%
Tumor grade			
	grade 1	2	13.3%
	grade 2	6	40.0%
	grade 3	6	40.0%
	not applicable	1	6.7%
Distant metastases			
	Yes	2	13.3%
	No	13	86.7%
Chemotherapy regimen			
	Anthracycline-based	7	46.7%
	Taxane-based	7	46.7%
	Others**	2	13.3%

*pT, pN stage: according to definition of AJCC 7th edition staging system.

**Others include: liposomal doxorubicin, vinorelbine. ER: estrogen receptor; PR: progesterone receptor; GCSF: Granulocyte colony-stimulating factor.

### Dynamic change of CEC, V-CEC, and CEP number after chemotherapy

**Figure 1:**
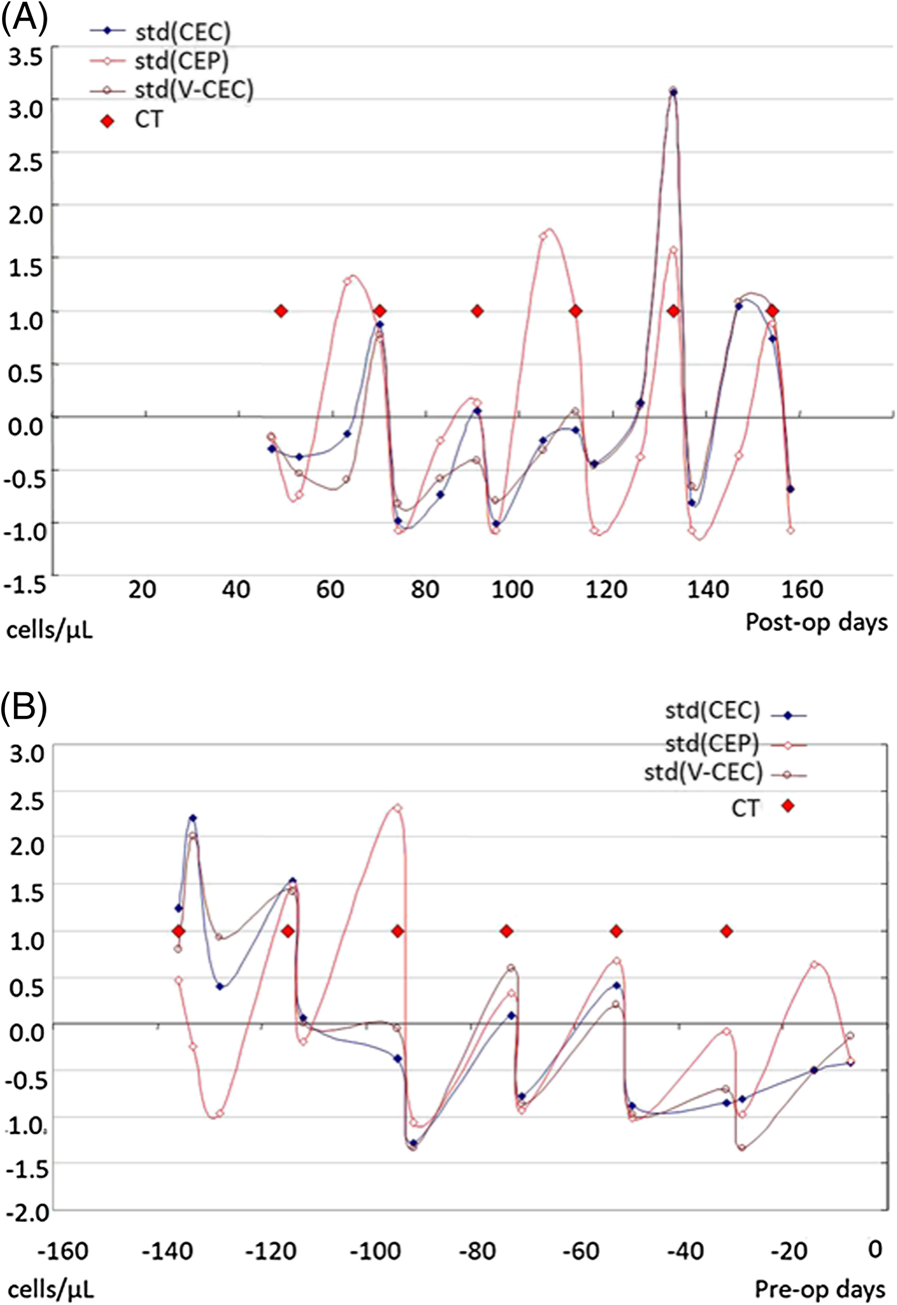
**Standardized trend of CEC, V-CEC, CEP, as a function of chemotherapy the day before or after tumor resection. (A)** Data from a non-tumor-bearing patient who received adjuvant chemotherapy after tumor resection. **(B)** Data from a tumor-bearing patient who received neoadjuvant chemotherapy before tumor resection. Chemotherapy immediately reduced the CEC, V-CEC, and CEP numbers, followed by a rebound elevation of the CEC and CEP numbers. The “wave” patterns were similar between the non-tumor-bearing and tumor-bearing patients.

Figure 1 illustrates trends in the levels of CEC, V-CEC, and CEP as a function of days of chemotherapy post-operation in one representative patient who was not tumor-bearing, and as a function of days of chemotherapy prior to operation in one patient who was tumor-bearing. The non-tumor-bearing patient (Figure 1A), a 60-year-old woman diagnosed with pT2N2aM0 stage IIIA breast cancer, received adjuvant TEC chemotherapy (paclitaxel, epirubicin, cyclophosphamide) for a total of six cycles after a modified radical mastectomy. The tumor-bearing patient (Figure 1B), a 56-year-old woman diagnosed with pT4bN2M0 stage IIIB breast cancer, received neoadjuvant TEC chemotherapy for a total of six cycles before a modified radical mastectomy. The changes in the levels of CEC and CEP of both patients had a similar wave pattern, with the chemotherapy immediately reducing the CEC and CEP levels followed by a rebound in their levels. Representative flow cytometry dot plots data of dynamic change of CEC, CEP and viable-CEC levels before and after chemotherapy from one patient was provided in our supplementary data. (Additional file 5: Figure S5).

**Figure 2:**
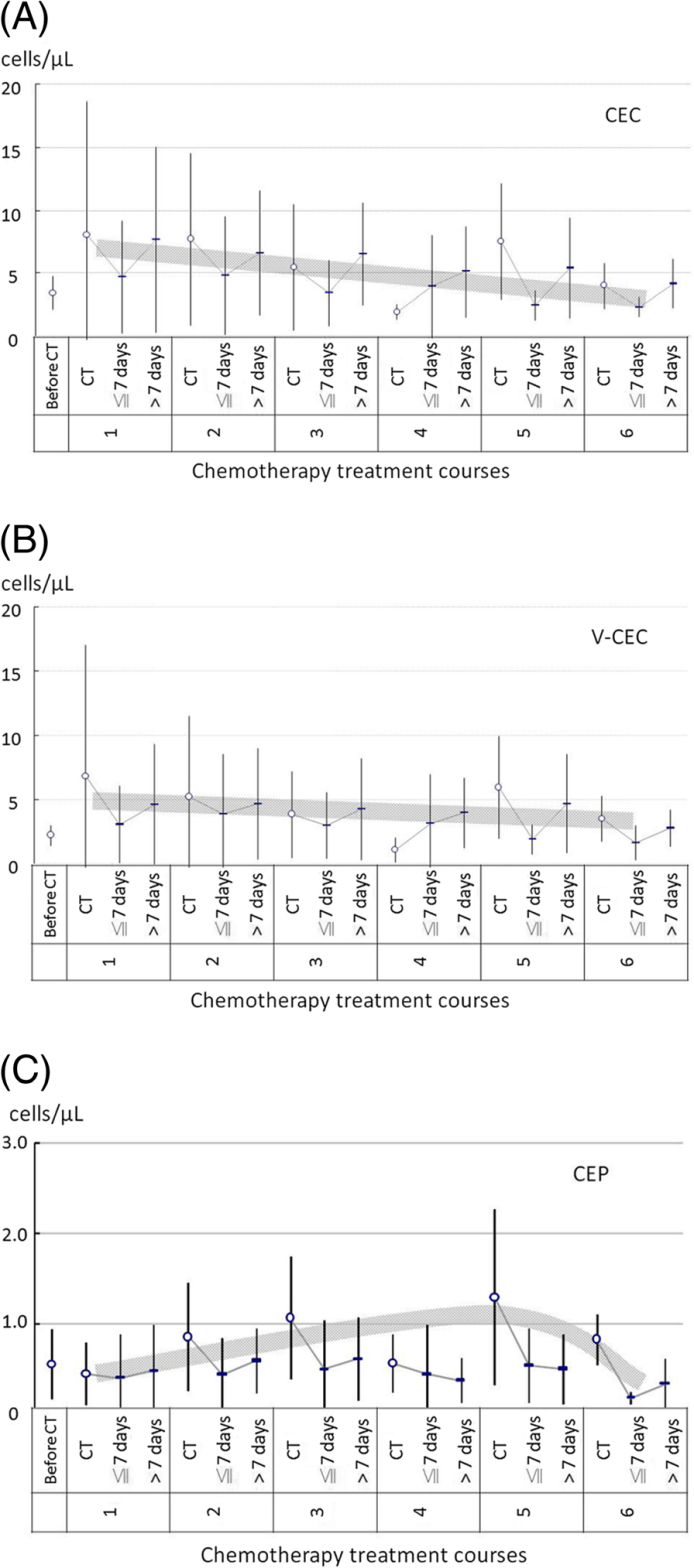
**The means and standard deviations of (A) CEC, (B) V-CEC, and (C) CEP along the course of treatment.** Within a chemotherapy course, the means of the CEC, V-CEC, and CEP were all significantly decreased in the 1^st^ week of chemotherapy as compared to the day chemotherapy started. After the 1^st^ week of treatment, the means of the CEC and V-CEC returned to a level close to that of the starting day of chemotherapy (Figure **2A**, **2B**). However, the mean of the CEP remained significantly reduced after the 1^st^ week of chemotherapy (Figure **2C**). Note that the CEP level gradually rebounded back to a level similar to or even higher than the day 1 count of the previous chemotherapy cycle (Figure **2C**). The gray shadow denotes the trends of CEC, V-CEC, and CEP among the courses of chemotherapy

During the first week of chemotherapy, the mean CEC levels decreased by -2.92/μL (95% CI = -4.93, -0.92), V-CEC by -2.29/μL (95% CI = -3.86, -0.72), and CEP by -0.37/μL (95% CI = -0.58, -0.15) compared to the day that chemotherapy started (Figure 2 and Table 2). After the first week of treatment, their levels of CEC and V-CEC returned to levels not significantly different from their levels on the first day of chemotherapy. However, the level of CEP remained significantly reduced after the first week of chemotherapy. On the first day of subsequent courses of chemotherapy, the CEP level gradually rebounded back to a level similar to or even higher than the first day of the previous cycle of chemotherapy (Figure 2C).

**Table 2:** CEC, V-CEC, CEP trends within and among the chemotherapy courses

**Trends within chemotherapy course**					
**Outcome measurement**	**Time point**	**Coefficient (cells/μL)**	**95% CI**	***p *value**	
CEC	start of CT	reference			
	1^st^ wk of CT	−2.924	(-4.93,−0.92)	< 0.01	
	after 1^st^ wk of CT	−0.909	(-2.80,0.98)	0.35	
	before 1^st^ CT	−5.848	(-9.83,−1.86)	< 0.01	
V-CEC	start of CT	reference			
	1^st^ wk of CT	−2.289	(-3.86,−0.72)	< 0.01	
	after 1^st^ wk of CT	−0.996	(-2.56,0.57)	0.21	
	before 1^st^ CT	−4.014	(-7.29,−0.73)	0.02	
CEP	start of CT	reference			
	1^st^ wk of CT	−0.366	(-0.58,−0.15)	< 0.01	
	after 1^st^ wk of CT	−0.295	(-0.47,−0.12)	< 0.01	
Trends among courses of chemotherapy					
Measurement	Terms	Coefficient (cells/μL)	95% CI	*p* value	
CEC	Linear	−0.609	(-1.12,-0.10)	0.02	
V-CEC	Linear	−0.288	(-0.72,0.15)	0.19	
CEP	Linear	0.194	(0.03,0.36)	0.02	
	Quadratic	−0.03	(-0.06,0.01)	0.02	

Before chemotherapy was first initiated, the mean levels CEC and V-CEC were significantly lower as compared to the initiation day of the 1^st^ course of chemotherapy (*P* < 0.01 and *P* < 0.02, respectively; Table 2). The level of CEC significantly declined (*P* = 0.02) and the V-CEC trended toward a decline over the courses of chemotherapy (gray shadows in Figure 2). The level of CEP gradually increased during the first three courses of chemotherapy and declined during the later treatment courses (Figure 2C). This “n”-shaped, convex trend was represented by a set of statistically significant positive linear (*P* = 0.02) and negative quadratic (*P* = 0.02) terms (Table 2).

The measurements were not significantly associated with factors including the presence of a tumor, the operation status, the drug used, and the use of GCSF (all *P* values > 0.1; Table 3).

**Table 3:** CEC, V-CEC, and CEP levels among different clinical conditions

	**CEC**		**V-CEC**		**CEP**	
**Variable**	Coef* (cells/μL)	***p *value**	**Coef (cells/μL)**	***p *value**	**Coef (cells/μL)**	***p *value**
Tumor bearing	−0.57	0.74	−1.08	0.46	0.10	0.44
Chemotherapy regimen*						
CEF	0.04	0.99	0.65	0.82	0.12	0.64
Liposomal doxorubicin	−3.00	0.58	−0.43	0.92	−0.03	0.93
N-HDFL	1.79	0.70	3.22	0.40	0.20	0.55
TCH	−1.42	0.63	−0.55	0.82	0.01	0.98
TEC	1.08	0.79	2.50	0.45	0.27	0.32
TH	(Ref)		(Ref)		(Ref)	
G-CSF use	0.03	0.85	−0.02	0.86	−0.004	0.78

*Coef = adjusted regression coefficient.

*CEF: cyclophosphamide, epirubicin, fluorouracil.

N-HDFL: vinorelbine, continuous infusion fluorouracil and leucovorin.

TCH: docetaxel, carboplatin, herceptin.

TEC: docetaxel, epirubicin, cyclophosphamide.

TH: docetaxel, herceptin.

## Discussion

We have clinically demonstrated the dynamic pattern of CEC and CEP during the course of chemotherapy treatment. The mean levels of CEC, V-CEC, and CEP all significantly decreased during the first week of chemotherapy. Then the levels of CEC and V-CEC returned gradually after one week whereas the level of CEP remained significantly reduced. After six cycles of chemotherapy, the level of CEC decreased significantly, V-CEC trended toward a decrease, and CEP increased significantly. These results were not significantly affected by clinical factors including the existence of a tumor, the chemotherapy regimen, and the use of GCSF.

The sole antigen to distinct CEPs with CECs is CD133 at present. However, human CD133 is not only expressed in endothelial progenitor cells but also in haematopoietic progenitor cells and bi-potential haemangioblasts. Currently, a phenotypic distinction between these three kinds of cells is not feasible and controversial [42]. In our six-color flow cytometry, CD45 expression can be used to exclude haematopoietic cells from the analysis. Theoretically, CD146 is expressed in endothelial cells but not platelet, and CD133 expression can be used to differentiate platelet and endothelial microparticles from CEP. However, lack of DNA staining is at a risk since a nuclear-staining molecule can be useful to exclude aggregated platelets and/or endothelial microparticles (which share some surface markers with CECs and CEPs) from the CEC count [42].

Longitudinal follow-up found that the levels of CEC and V-CEC increased from the baseline before chemotherapy to the first day of the first cycle of chemotherapy, but this did not occur with the CEPs (Figure 2). Current thought is that CECs are mature endothelial cells that have detached from their basement membrane in response to a blood vessel injury [24,42]. The increase in CEC and V-CEC levels we observed may be due to vessel damage caused by implanting port-A catheters or by tumor resection surgery, since all of the patients were treated with one of these two procedures before the first day of the first cycle of chemotherapy. The CEPs are progenitor cells that are recruited from bone marrow rather than detached from the vessel wall, and their level did not change in a similar manner.

Since several studies have indicated that levels of CEC and CEP correlate with tumor size and grade, [20,40,43-46] we assumed that tumor-bearing patients would have higher CEC and CEP levels than patients without tumors, but our analysis found no significant differences. Perhaps CEC and CEP levels correlate with not just the presence of a tumor, but with many factors, including tumor size, tumor grade, vascular invasion, and lymphatic invasion. Although patients who received adjuvant chemotherapy mostly had no gross residual tumor, but vascular or lymphatic invasion, even cancer related cytokines was possible and may explain for the CEC and CEP kinetics. Since our analysis of CEC and CEP levels in tumor–bearing and non–tumor bearing patients included a mix of many variables, we cannot make a conclusion about which variables affect their levels. Here we provided some representative figures of the CEC and CEP kinetics after administration of chemotherapy in tumor-bearing and non-tumor bearing patients (Additional file 6: Figure S6). The CEC and CEP kinetics consistently showed similar wave pattern and had no obvious differences between patients with and without tumor bearing. This explanation is also consistent with a study that found that CEC and CEP levels did not significantly change before and after surgery in 15 breast cancer patients, although vascular invasion and tumor size were independently associated with the CEC levels [43].

Administering GCSF to mice has been found to elevate CEP and accelerate tumor growth [47-49]. A human study analyzing CEC levels in four patients (one with seminoma, two with nonseminoma testis cancer, and one with small-cell lung cancer) receiving chemotherapy with the support of GCSF found that three out of four patients had CEC amounts increased 8- to 9-fold 3–8 days after GCSF administration [50]. In the same study, the CEC level was almost undetectable in leukopenic patient. This implies that the GCSF effect on CEC levels may be markedly related to bone marrow recovery after chemotherapy. When we compared the seven patients who received GCSF during chemotherapy courses to the eight who did not, we found no differences in the dynamic trends in the CEC and CEP levels between these groups. Since our patients received different chemotherapy regimens and may have different grades of bone marrow suppression which lead to different timing of bone marrow recovery. We suggest that in our study, the effects of chemotherapy on CEC and CEP may mask the GCSF effect.

We wanted to understand whether CEC and CEP levels are affected differently by different chemotherapy agents. Certain chemotherapy drugs, such as taxane and fluorouracil, have been demonstrated to rapidly induce CEP mobilization and subsequent tumor homing, while others, such as gemcitabine, cisplatin, and doxorubicin, do not [37]. Our analysis of 29 cycles of taxane-based chemotherapy and 26 cycles of non-taxane-based chemotherapy found no significant differences in the dynamic trends of these two groups. However, this is not conclusive, since all of the chemotherapy regimens in this study used multiple chemotherapy agents and it is difficult to evaluate the effect of single chemotherapy agent on CEC/CEP kinetics. We provided some representative figures of the CEC and CEP kinetics after administration of different types of chemotherapy regimens in five different patients (Additional file 7: Figure S7). Their CEC and CEP kinetics consistently show similar wave pattern. It suggests that dynamic changes of CEC and CEP induced by chemotherapy may have more significant effect than using different drugs. It supports our conclusion that timing of measurement of CEC and CEP after chemotherapy should be seriously considered and unified in the future studies using CEC and CEP as endpoint.

The levels of CEC and V-CEC significantly decreased as the number of chemotherapy courses progressed (*P* = 0.02), but the CEP levels significantly increased during the first three courses of chemotherapy (*P* = 0.02) and then declined during later treatment courses. Although this study was restricted by the small number of patients and chemotherapy cycles, the trends we observed were consistent with the analysis of Furstenberger et al. [39]. They analyzed the levels of CEC and CEP before and after neoadjuvant chemotherapy in breast cancer patients and found that, after two cycles of chemotherapy, the CEC levels decreased and the CEP levels increased. This phenomenon may be explained by the CEPs behaving as progenitor cells that could be mobilized from the bone marrow by a regular dose of chemotherapy. Although the CEPs would gradually differentiate into CECs, the next course of chemotherapy might destroy some cells that were in transition before they could fully differentiate into CECs. This may explain why CEC levels decreased and CEP levels increased after cycles of chemotherapy.

Many preclinical studies have indicated that CEPs contribute to tumor growth [23,40,43], which warrants further concern that chemotherapy may remobilize CEPs and trigger additional tumor growth. We demonstrated that the CEC and CEP levels decreased in the first week of chemotherapy, CEC increased after one week with each cycle of chemotherapy, and CEP rebounded even more slowly than CEC. This suggests to us that the rate of tumor control achieved by chemotherapy may theoretically be helped by adding metronomic chemotherapy or an antiangiogenic agent one week after chemotherapy to suppress the rebound of CEC and CEP. This hypothesis warrants further prospective study.

Our observation that CEC rebounds earlier than CEP has two possible explanations: 1) CEP is not the only source of CEC, which has three main sources: tumor vasculature, normal vasculature, and CEP. The three sources could explain why CEC is not fully synchronized with CEP. 2) The CEPs are a kind of progenitor cell where one CEP may differentiate into a population of CECs, which would explain the earlier rise in the slope of the CECs as compared to the CEPs.

## Conclusions

The levels of CEC and CEP change dynamically during and after each course of chemotherapy. Careful selection of the timing of sample collection in each chemotherapy cycle is needed when using CEC and CEP as surrogate markers of angiogenesis.

## Competing interests

The authors declare that they have no competing interests.

## Authors’ contributions

Yu-Hsuan Kuo, Ching-Hung Lin, Wen-Yee Shau, Te-Jung Chen, Shih-Hung Yang, Shu-Min Huang, Chun Hsu, Yen-Shen Lu, Ann-Lii Cheng. AL Cheng, YS Lu and Chin-Hung Line participated in the design of the study. TJ Chen and SM Huang carried out the six-color flow cytometry. WY Shau performed the statistical analysis. SH Yang and C Hsu contribute to acquisition of data. YH Kuo integrate the data and help to draft the manuscript. All authors read and approved the final manuscript.

## Pre-publication history

The pre-publication history for this paper can be accessed here:

http://www.biomedcentral.com/1471-2407/12/620/prepub

## Supplementary Material

Additional file 1: Figure S1. CEC and CEP test were preformed within 24 hours of collection of blood samples. The gating strategy is described below. Exclude debris and red blood cells first. CEC and CEPs are within CD45dim population (P2). CD31/CD146 double positive population (Q2) were defined as CECs (S-Figure 1 and 2). Both CD31/CD133 (Q2-1) and CD146/CD133 (Q2-2) double positive were CEPs (S-Figure 1 and 2). CEPs number presented here were the average of Q2-1 dot number and Q2-2 dot number. For gating viable-CEC, excluding debris and red blood cells first and CEC and CEPs are within CD45dim population (P2). CD146 was CEC maker and 7AAD staining was used to identify the cell viability. Cells in Q2 are apoptotic CECs and in Q4 are viable CECs. (S-Figure 3 and 4) Unstain sample was used as a negative control (S-Figure 1 and 3). CEC/CEP and viable CEP gating was follow the unstain one. (S-Figure 2 and 4). Click here for file

Additional file 2: Figure S2. CEC and CEP test were preformed within 24 hours of collection of blood samples. The gating strategy is described below. Exclude debris and red blood cells first. CEC and CEPs are within CD45dim population (P2). CD31/CD146 double positive population (Q2) were defined as CECs (S-Figure 1 and 2). Both CD31/CD133 (Q2-1) and CD146/CD133 (Q2-2) double positive were CEPs (S-Figure 1 and 2). CEPs number presented here were the average of Q2-1 dot number and Q2-2 dot number. For gating viable-CEC, excluding debris and red blood cells first and CEC and CEPs are within CD45dim population (P2). CD146 was CEC maker and 7AAD staining was used to identify the cell viability. Cells in Q2 are apoptotic CECs and in Q4 are viable CECs. (S-Figure 3 and 4) Unstain sample was used as a negative control (S-Figure 1 and 3). CEC/CEP and viable CEP gating was follow the unstain one. (S-Figure 2 and 4). Click here for file

Additional file 3: Figure S3. CEC and CEP test were preformed within 24 hours of collection of blood samples. The gating strategy is described below. Exclude debris and red blood cells first. CEC and CEPs are within CD45dim population (P2). CD31/CD146 double positive population (Q2) were defined as CECs (S-Figure 1 and 2). Both CD31/CD133 (Q2-1) and CD146/CD133 (Q2-2) double positive were CEPs (S-Figure 1 and 2). CEPs number presented here were the average of Q2-1 dot number and Q2-2 dot number. For gating viable-CEC, excluding debris and red blood cells first and CEC and CEPs are within CD45dim population (P2). CD146 was CEC maker and 7AAD staining was used to identify the cell viability. Cells in Q2 are apoptotic CECs and in Q4 are viable CECs. (S-Figure 3 and 4) Unstain sample was used as a negative control (S-Figure 1 and 3). CEC/CEP and viable CEP gating was follow the unstain one. (S-Figure 2 and 4). Click here for file

Additional file 4: Figure S4. CEC and CEP test were preformed within 24 hours of collection of blood samples. The gating strategy is described below. Exclude debris and red blood cells first. CEC and CEPs are within CD45dim population (P2). CD31/CD146 double positive population (Q2) were defined as CECs (S-Figure 1 and 2). Both CD31/CD133 (Q2-1) and CD146/CD133 (Q2-2) double positive were CEPs (S-Figure 1 and 2). CEPs number presented here were the average of Q2-1 dot number and Q2-2 dot number. For gating viable-CEC, excluding debris and red blood cells first and CEC and CEPs are within CD45dim population (P2). CD146 was CEC maker and 7AAD staining was used to identify the cell viability. Cells in Q2 are apoptotic CECs and in Q4 are viable CECs. (S-Figure 3 and 4) Unstain sample was used as a negative control (S-Figure 1 and 3). CEC/CEP and viable CEP gating was follow the unstain one. (S-Figure 2 and 4). Click here for file

Additional file 5: Figure S5. Representative flow cytometry dot plot for defining viable CECs and apoptotic CECs, (A) Exclude debris and red blood cells. (B) CEC and CEPs are within CD45dim population (P2). (C)CD146 was CEC maker and 7AAD staining was used to identify the cell viability. Cells in Q2 are apoptotic CECs and in Q4 are viable CECsRepresentative data of dynamic change of CEC, CEP (S-Figure 5a, 5c, 5e and 5 g) and viable-CEC (S-Figure 5b, 5d, 5f and 5 h) levels during second cycle of chemotherapy from one patient. CEC, CEP (S-Figure 5a) and viable-CEC (S-Figure 5b) at the day before chemotherapy (for this patient, taxotere /epirubicin /cyclophosphamide) were shown in S-Figure 5a and 5b. Patient’s CEC, CEP and viable-CEC levels were dropping at day 4 and day 7 (S-Figure 5c, 5d, 5e and 5f) after chemotherapy. Three weeks after chemotherapy, CEC and CEP levels were increased again(S-Figure 5e) and most of the CECs were viable (S-Figure 5f). Click here for file

Additional file 6: Figure S6. Standardized trend of CEC, V-CEC, CEP, as a function of chemotherapy the day before tumor resection (A-D), or after tumor resection.(E-G). The CEC and CEP kinetics consistently showed similar wave pattern and had no obvious differences between patients with and without tumor bearing. Click here for file

Additional file 7: Figure S7. Standardized trend of CEC, V-CEC, CEP, as a function of different chemotherapy regimens. (A) Data from a patient who received adjuvant docetaxel, cisplatin, and herceptin. (B) Data from a patient who received adjuvant cyclophosphamide, epirubicin, and Fluorouracil. (C) Data from a patient who received adjuvant docetaxel, epirubicin, and cyclophophamide. (D) Data from a patient who received neoadjuvant docetaxel, Epirubicin, and cyclophophamide. (E) Data from a patient who received neoadjuvant vinorelbine and infusion fluorouracil. Their CEC and CEP kinetics consistently show similar wave pattern. It suggests that dynamic changes of CEC and CEP induced by chemotherapy may have more significant effect than using different drugs. Click here for file
