# Supplementary figures and images for: Dynamics of circulating endothelial cells and endothelial progenitor cells in breast cancer patients receiving cytotoxic chemotherapy
Source: BMC Cancer. 2012 Dec 26;12:620. doi: 10.1186/1471-2407-12-620 (PMC3561193; doi:10.1186/1471-2407-12-620)

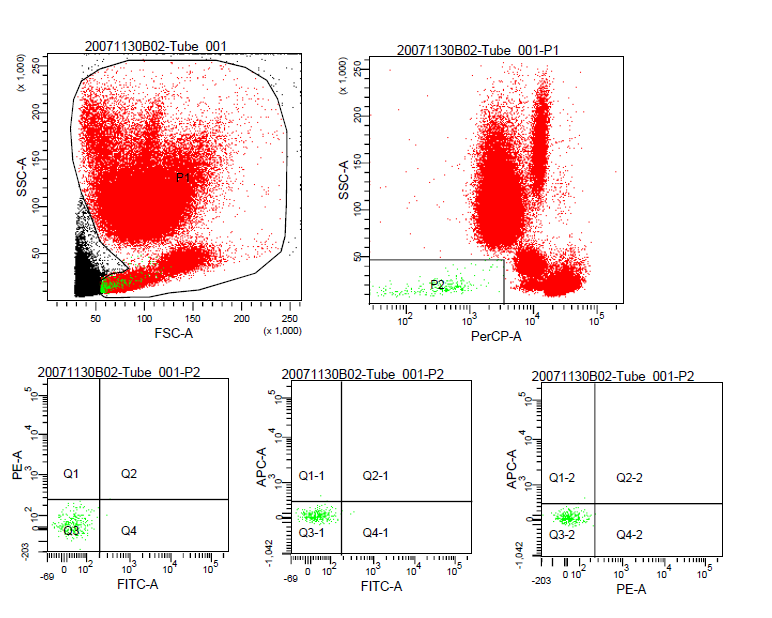


Figure 1

CD45

CD 31

CD 146

CD 31

CD 133

CD 146

CD 133

Supplement: Additional file 1: Figure S1. — CEC and CEP test were preformed within 24 hours of collection of blood samples. The gating strategy is described below. Exclude debris and red blood cells first. CEC and CEPs are within CD45dim population (P2). CD31/CD146 double positive population (Q2) were defined as CECs (S-Figure 1 and 2). Both CD31/CD133 (Q2-1) and CD146/CD133 (Q2-2) double positive were CEPs (S-Figure 1 and 2). CEPs number presented here were the average of Q2-1 dot number and Q2-2 dot number. For gating viable-CEC, excluding debris and red blood cells first and CEC and CEPs are within CD45dim population (P2). CD146 was CEC maker and 7AAD staining was used to identify the cell viability. Cells in Q2 are apoptotic CECs and in Q4 are viable CECs. (S-Figure 3 and 4) Unstain sample was used as a negative control (S-Figure 1 and 3). CEC/CEP and viable CEP gating was follow the unstain one. (S-Figure 2 and 4). [file 1471-2407-12-620-S1.docx]

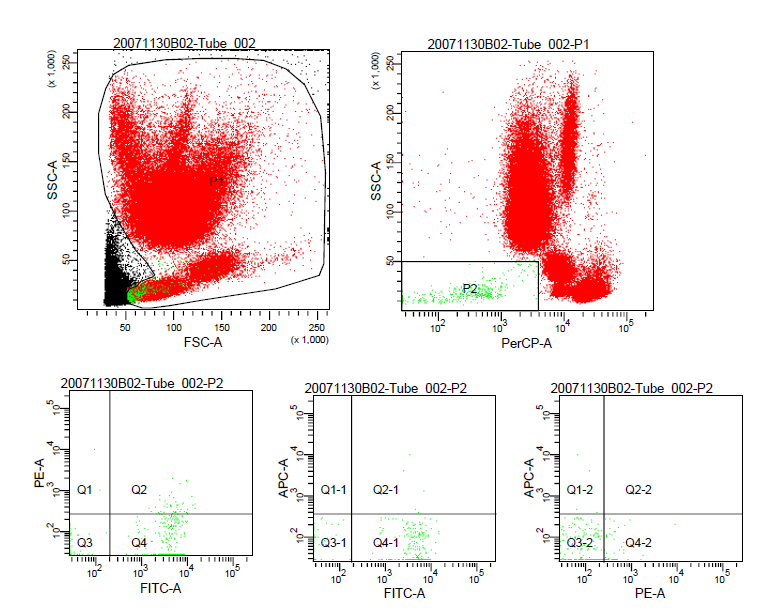


Figure 2

CD45

CD 31

CD 146

CD 133

CD 31

CD 146

CD 133

Supplement: Additional file 2: Figure S2. — CEC and CEP test were preformed within 24 hours of collection of blood samples. The gating strategy is described below. Exclude debris and red blood cells first. CEC and CEPs are within CD45dim population (P2). CD31/CD146 double positive population (Q2) were defined as CECs (S-Figure 1 and 2). Both CD31/CD133 (Q2-1) and CD146/CD133 (Q2-2) double positive were CEPs (S-Figure 1 and 2). CEPs number presented here were the average of Q2-1 dot number and Q2-2 dot number. For gating viable-CEC, excluding debris and red blood cells first and CEC and CEPs are within CD45dim population (P2). CD146 was CEC maker and 7AAD staining was used to identify the cell viability. Cells in Q2 are apoptotic CECs and in Q4 are viable CECs. (S-Figure 3 and 4) Unstain sample was used as a negative control (S-Figure 1 and 3). CEC/CEP and viable CEP gating was follow the unstain one. (S-Figure 2 and 4). [file 1471-2407-12-620-S2.docx]

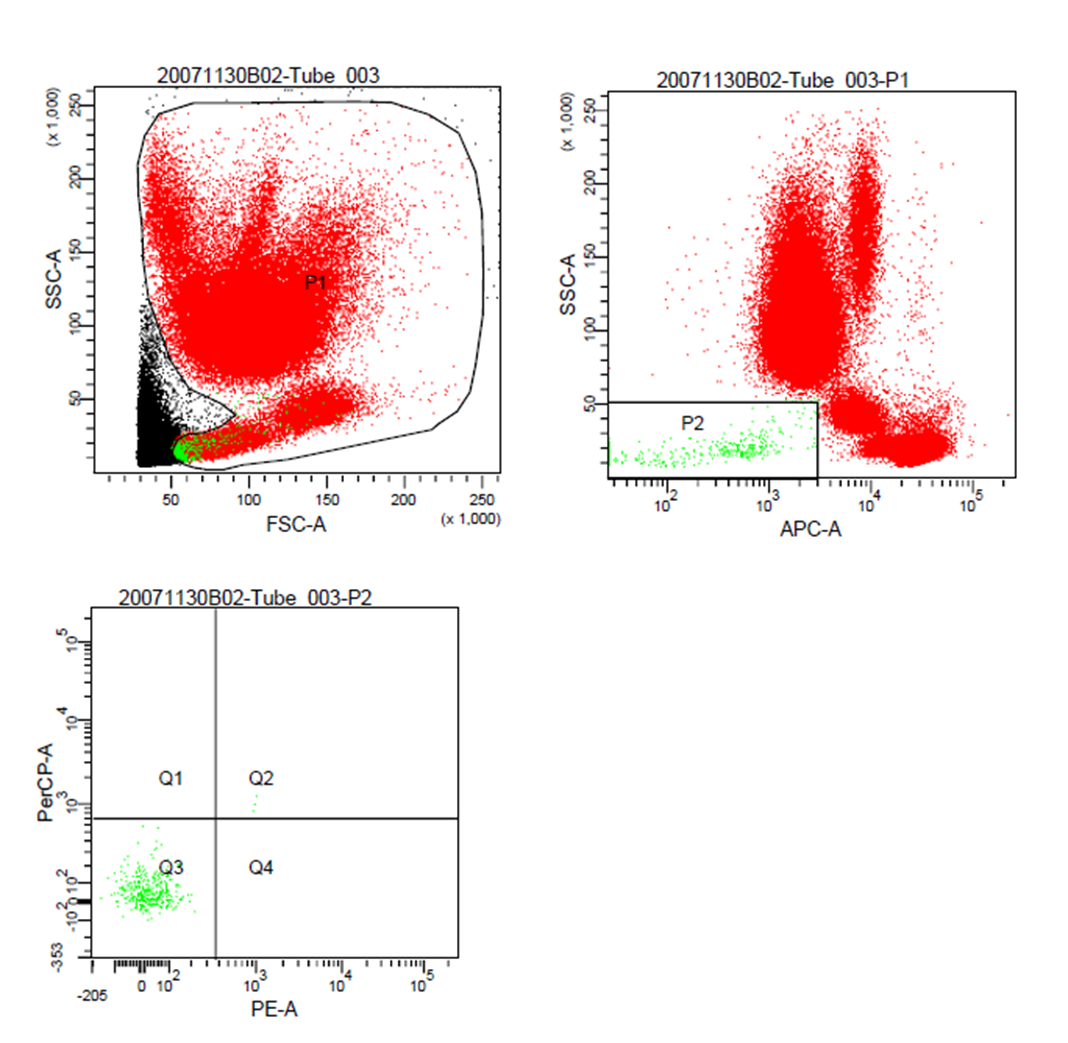


7AAD

CD146

CD45

Supplement: Additional file 3: Figure S3. — CEC and CEP test were preformed within 24 hours of collection of blood samples. The gating strategy is described below. Exclude debris and red blood cells first. CEC and CEPs are within CD45dim population (P2). CD31/CD146 double positive population (Q2) were defined as CECs (S-Figure 1 and 2). Both CD31/CD133 (Q2-1) and CD146/CD133 (Q2-2) double positive were CEPs (S-Figure 1 and 2). CEPs number presented here were the average of Q2-1 dot number and Q2-2 dot number. For gating viable-CEC, excluding debris and red blood cells first and CEC and CEPs are within CD45dim population (P2). CD146 was CEC maker and 7AAD staining was used to identify the cell viability. Cells in Q2 are apoptotic CECs and in Q4 are viable CECs. (S-Figure 3 and 4) Unstain sample was used as a negative control (S-Figure 1 and 3). CEC/CEP and viable CEP gating was follow the unstain one. (S-Figure 2 and 4). [file 1471-2407-12-620-S3.docx]

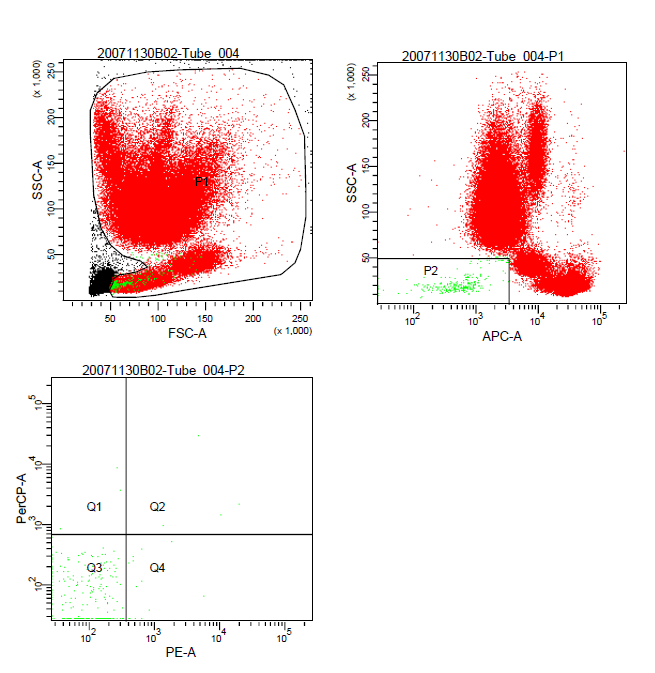


CD146

CD45

7AAD

Supplement: Additional file 4: Figure S4. — CEC and CEP test were preformed within 24 hours of collection of blood samples. The gating strategy is described below. Exclude debris and red blood cells first. CEC and CEPs are within CD45dim population (P2). CD31/CD146 double positive population (Q2) were defined as CECs (S-Figure 1 and 2). Both CD31/CD133 (Q2-1) and CD146/CD133 (Q2-2) double positive were CEPs (S-Figure 1 and 2). CEPs number presented here were the average of Q2-1 dot number and Q2-2 dot number. For gating viable-CEC, excluding debris and red blood cells first and CEC and CEPs are within CD45dim population (P2). CD146 was CEC maker and 7AAD staining was used to identify the cell viability. Cells in Q2 are apoptotic CECs and in Q4 are viable CECs. (S-Figure 3 and 4) Unstain sample was used as a negative control (S-Figure 1 and 3). CEC/CEP and viable CEP gating was follow the unstain one. (S-Figure 2 and 4). [file 1471-2407-12-620-S4.docx]

1. (b)


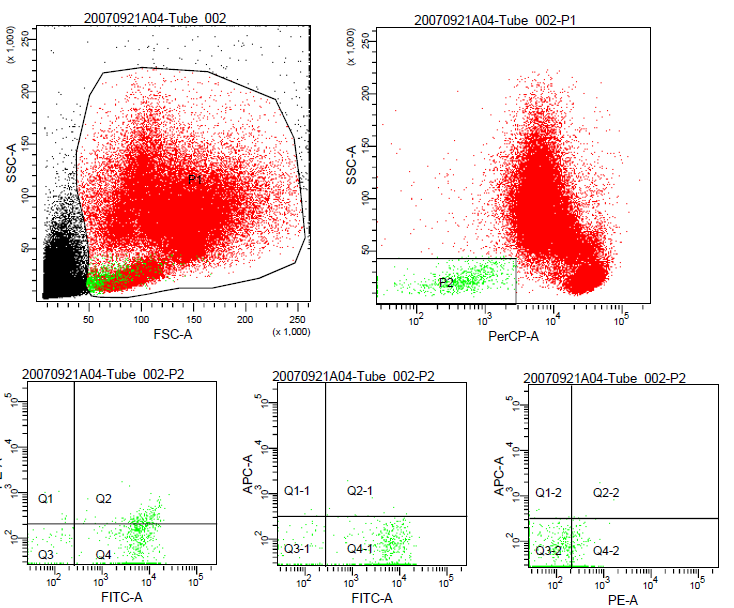

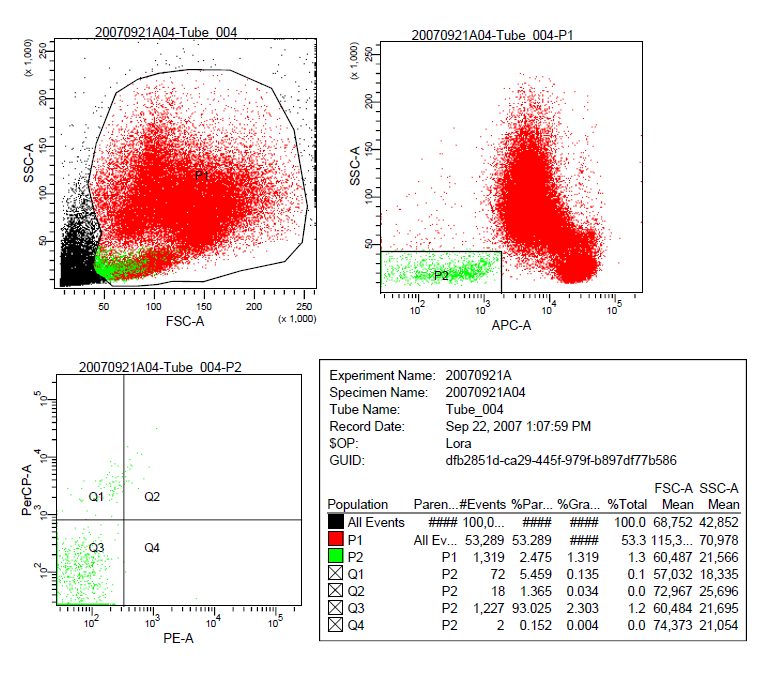


CD 45

CD 146

CD 45

CD 146

CD 31

CD 31

(c) (d)


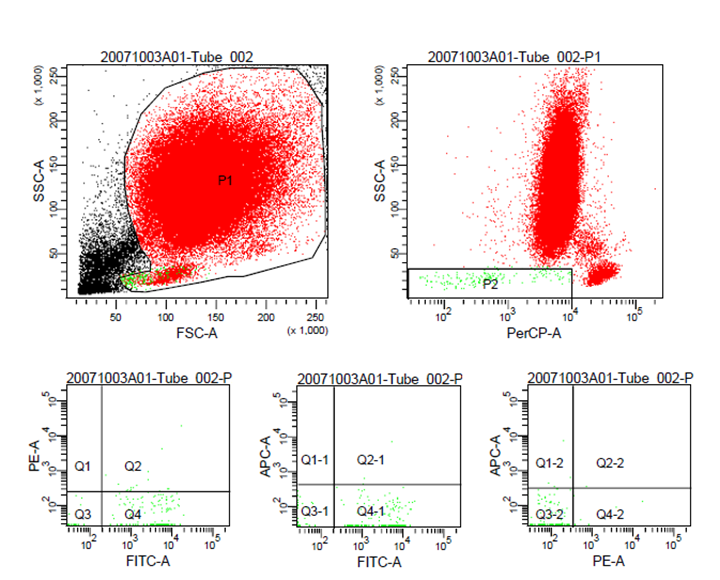

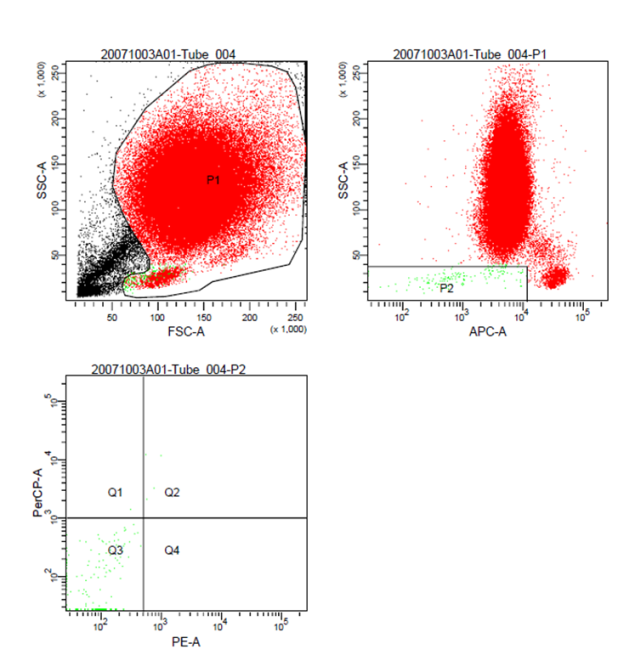


CD 146

CD 146

CD 31

CD 31

CD 45

CD 45

(e) (f)


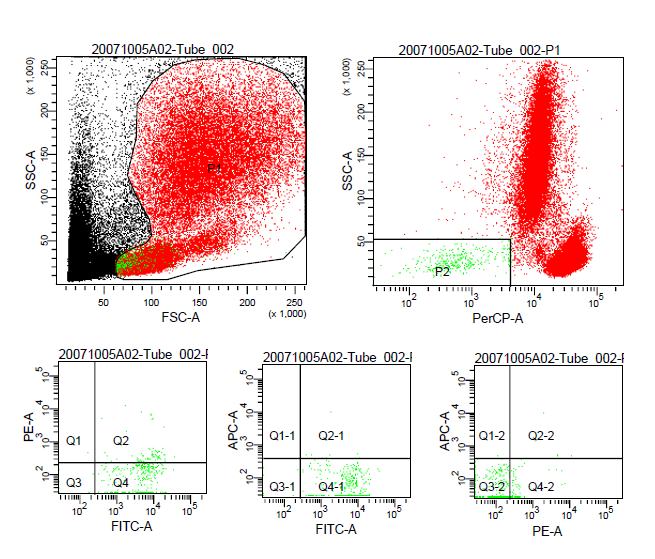

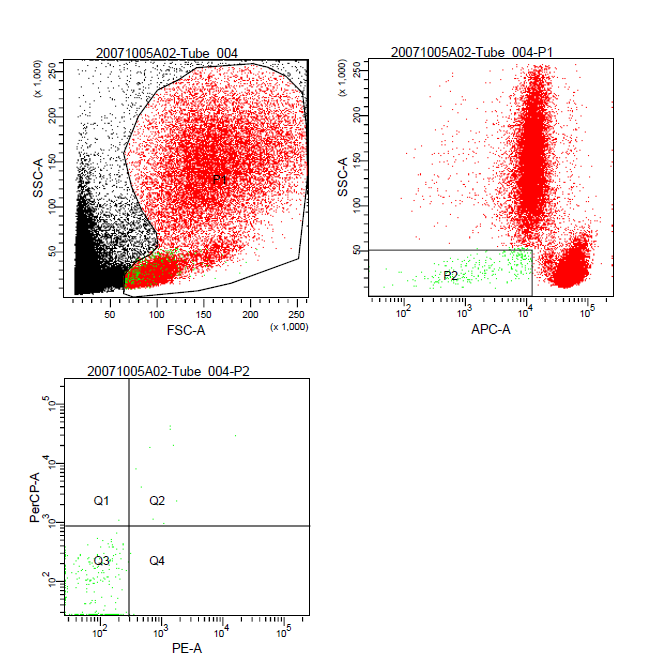


CD 31

CD 31

CD 45

CD 45

CD 146

CD 146

(g) (h)


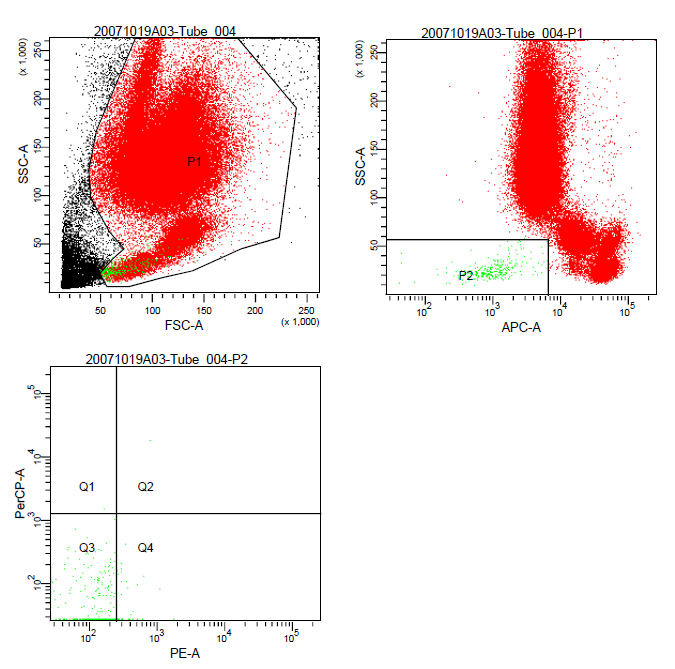

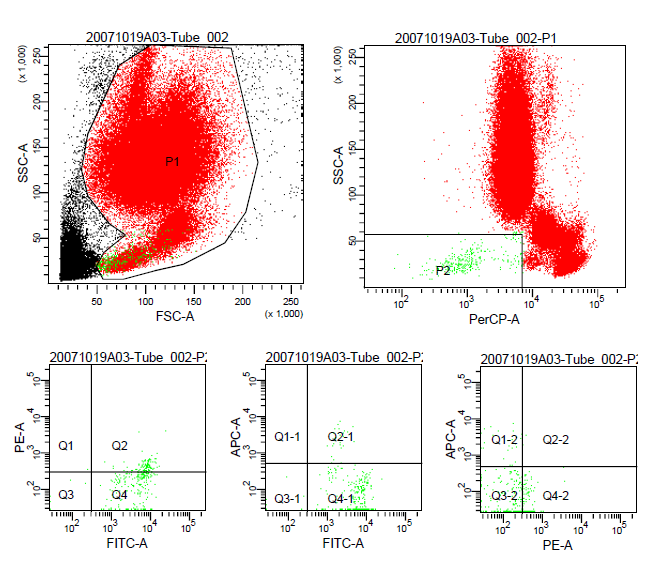


CD 31

CD 31

CD 45

CD 45

CD 146

CD 146

Supplement: Additional file 5: Figure S5. — Representative flow cytometry dot plot for defining viable CECs and apoptotic CECs, (A) Exclude debris and red blood cells. (B) CEC and CEPs are within CD45dim population (P2). (C)CD146 was CEC maker and 7AAD staining was used to identify the cell viability. Cells in Q2 are apoptotic CECs and in Q4 are viable CECsRepresentative data of dynamic change of CEC, CEP (S-Figure 5a, 5c, 5e and 5 g) and viable-CEC (S-Figure 5b, 5d, 5f and 5 h) levels during second cycle of chemotherapy from one patient. CEC, CEP (S-Figure 5a) and viable-CEC (S-Figure 5b) at the day before chemotherapy (for this patient, taxotere /epirubicin /cyclophosphamide) were shown in S-Figure 5a and 5b. Patient’s CEC, CEP and viable-CEC levels were dropping at day 4 and day 7 (S-Figure 5c, 5d, 5e and 5f) after chemotherapy. Three weeks after chemotherapy, CEC and CEP levels were increased again(S-Figure 5e) and most of the CECs were viable (S-Figure 5f). [file 1471-2407-12-620-S5.docx]
