# Supplementary material for: Dynamics of circulating endothelial cells and endothelial progenitor cells in breast cancer patients receiving cytotoxic chemotherapy
Source: BMC Cancer. 2012 Dec 26;12:620. doi: 10.1186/1471-2407-12-620 (PMC3561193; doi:10.1186/1471-2407-12-620)
Supplement: Additional file 6: Figure S6. — Standardized trend of CEC, V-CEC, CEP, as a function of chemotherapy the day before tumor resection (A-D), or after tumor resection.(E-G). The CEC and CEP kinetics consistently showed similar wave pattern and had no obvious differences between patients with and without tumor bearing. [file 1471-2407-12-620-S6.docx]

(A)

Cells/μL

Post-op days

(B)

Cells/μL

Post-op days

(C)

Cells/μL

Post-op days

(D)

Cells/μL

Post-op days

(E)

Cells/μL

Post-op days

(F)

Cells/μL

Post-op days

(G)

Cells/μL

Post-op days
