# Supplementary material for: Dynamics of circulating endothelial cells and endothelial progenitor cells in breast cancer patients receiving cytotoxic chemotherapy
Source: BMC Cancer. 2012 Dec 26;12:620. doi: 10.1186/1471-2407-12-620 (PMC3561193; doi:10.1186/1471-2407-12-620)
Supplement: Additional file 7: Figure S7. — Standardized trend of CEC, V-CEC, CEP, as a function of different chemotherapy regimens. (A) Data from a patient who received adjuvant docetaxel, cisplatin, and herceptin. (B) Data from a patient who received adjuvant cyclophosphamide, epirubicin, and Fluorouracil. (C) Data from a patient who received adjuvant docetaxel, epirubicin, and cyclophophamide. (D) Data from a patient who received neoadjuvant docetaxel, Epirubicin, and cyclophophamide. (E) Data from a patient who received neoadjuvant vinorelbine and infusion fluorouracil. Their CEC and CEP kinetics consistently show similar wave pattern. It suggests that dynamic changes of CEC and CEP induced by chemotherapy may have more significant effect than using different drugs. [file 1471-2407-12-620-S7.docx]

(A)

Cells/μL

Post-op days

(B)

Cells/μL

Post-op days

(C)

Cells/μL

Post-op days

(D)

Cells/μL

Post-op days

(E)

Cells/μL

Post-op days
